# Possible Evidence for Berezinskii–Kosterlitz–Thouless Transition in Ba(Fe_0.914_Co_0.086_)_2_As_2_ Crystals

**DOI:** 10.3390/ma14216294

**Published:** 2021-10-22

**Authors:** Wen-He Jiao, Xiao-Feng Xu, Hao Jiang, Zhu-An Xu, Qing-Hu Chen, Guang-Han Cao

**Affiliations:** 1 Interdisciplinary Center for Quantum Information, Zhejiang Province Key Laboratory of Quantum Technology and Devices, Department of Physics, Zhejiang University, Hangzhou 310027, China; zhuan@zju.edu.cn (Z.-A.X.); qhchen@zju.edu.cn (Q.-H.C.); 2 Ningbo Institute of Materials Technology and Engineering, Chinese Academy of Sciences, Ningbo 315201, China; 3 Department of Applied Physics, Zhejiang University of Technology, Hangzhou 310023, China; xuxiaofeng@zjut.edu.cn; 4 School of Physics and Optoelectronics, Xiangtan University, Xiangtan 411105, China; jialay@zju.edu.cn

**Keywords:** Berezinskii–Kosterlitz–Thouless transition, superconducting transition, vortices

## Abstract

In this study, we measure the in-plane transport properties of high-quality Ba(Fe_0.914_Co_0.086_)_2_As_2_ single crystals. Signatures of vortex unbinding Berezinskii–Kosterlitz–Thouless (BKT) transition are shown from both the conventional approach and the Fisher–Fisher–Huse dynamic scaling analysis, in which a characteristic Nelson–Kosterlitz jump is demonstrated. We also observe a non-Hall transverse signal exactly at the superconducting transition, which is explained in terms of guided motion of unbound vortices.

## 1. Introduction

Uncovering the underlying essential universality for high-temperature superconductivity is extremely important for understanding the superconducting mechanism as well as exploring the next high-*T*_c_ materials [1]. The two classes of high-temperature superconductors discovered, Cu-based superconductors (CuSCs) and Fe-based superconductors (FeSCs), bear many similarities, regardless of some differences [1,2,3]. Apparently, FeSCs contain two-dimensional (2D) FeAs layers, analogous to the 2D CuO_2_ planes in CuSCs. More similarities were manifested by the antiferromagnetism in parent compounds, superconducting phase diagrams with respect to chemical doping, and extreme type-II superconductivity with very high upper critical field, etc. However, FeSCs show unusually small anisotropy in the upper critical field [4,5], which is in sharp contrast with CuSCs [6]. So far, there is no consensus on the nature of dimensionality in FeSCs [1], unlike the situation in CuSCs where the 2D characteristic is widely observed, [6] and the 2D nature is considered to be crucial for high-*T*_c_ superconductivity [7].

Quasi-2D superconducting behaviors in CuSCs are demonstrated by a 2D Berezinskii–Kosterlitz–Thouless (BKT) topological phase transition [8] close to the mean-field superconducting transition temperature ($T_{c}^{\mathrm{MF}}$), even for the bulk crystals [9,10,11,12,13]. Such a novel BKT-type transition was earlier discussed in terms of vortex–antivortex dissociation in an ideal 2D superconductor [14], and then it was experimentally observed in ultra-thin superconducting films [15]. For CuSCs, because of the negligibly weak interlayer superconducting coupling near $T_{c}^{\mathrm{MF}}$, vortex fluctuations [16], or thermal distortions [17] in Josephson coupled layered materials, 2D BKT behavior was able to be observed in bulk crystals of Bi_2_Sr_2_CaCu_2_O_8_ [9,10], YBa_2_Cu_3_O_7_ [11,12], and La_1.875_Ba_0.125_CuO_4_ [13]. The expected unbound free vortices near the superconducting transition are independently supported by the observation of non-zero transverse voltage at zero field (hereafter denoted as $V_{0}^{xy}$) [18,19]. The 2D superconducting phase fluctuations are also evidenced even well above the superconducting transition temperature [20], shedding light on the superconducting mechanism of CuSCs.

As for FeSCs, either 2D or 3D of the nature of superconducting fluctuations is reported [21,22,23,24,25,26], and an apparent contradiction appeared in a few cases. For example, 2D nature of superconductivity was implied by the study of fluctuation conductivity in F-doped SmFeAsO polycrystals [21] and single crystals [22], while fluctuations in SmFeAsO_0.8_F_0.2_ was reported to have a 3D character and extend far above *T*_c_ [24]. Recently, the evidence of BKT transition was reported in FeTe_0.55_Se_0.45_ thin films, suggesting a quasi-2D characteristic in such systems [27]. This finding motivates us to explore possible BKT transition in other FeSCs. In this study, we report possible evidence for BKT phase transition in a typical FeSC, Ba(Fe_1−*x*_Co*_x_*)_2_As_2_ [28] with *x* = 0.086, via both the conventional approach and the Fisher–Fisher–Huse (FFH) [29] dynamic scaling analysis. The characteristic Nelson–Kosterlitz jump for a BKT transition is demonstrated. In addition, we observe non-Hall-type transverse signal including $V_{0}^{xy}$, exactly above the possible BKT transition temperature *T*_BKT_, pointing to the existence of thermally excited unbound vortices.

## 2. Experimental Methods

**Figure 1:**
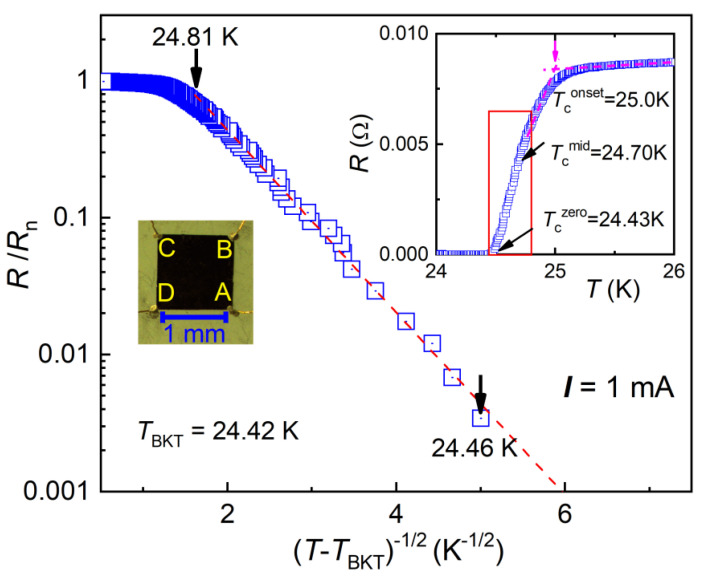
Temperature dependence of normalized resistance *R/R*_n_ in the superconducting transition of Ba(Fe_0.914_Co_0.086_)_2_As_2_ crystal (photographed on the left inset). Rn is the normal-state resistance, obtained by a linear extrapolation from 50 K to 35 K. The inserted plot shows the superconducting transition in normal linear scale. The related characteristic temperatures are indicated with arrows.

The Ba(Fe_0.914_Co_0.086_)_2_As_2_ crystals were grown by a self-flux method with procedures similar to Ref. [30]. The chemical composition of the crystal was determined by an energy-dispersive x-ray spectroscope affiliated to a field-emission scanning electron microscope (FEI Model SIRION), giving the title chemical formula (the Co-content uncertainty was ±0.005). The $T_{c}^{\mathrm{onset}}$ temperature-dependent in-plane resistance shows a sharp superconducting transition at $T_{c}^{\mathrm{onset}}$= 25.0 K, as seen in the inset of Figure 1. The transition width [Δ*T*_c_ = *T*(90%*ρ*_n_) − *T*(10%*ρ*_n_), where *ρ*_n_ is the normal-state resistivity at $T_{c}^{\mathrm{onset}}$] is as narrow as 0.42 K, indicating high quality with good homogeneity for the crystal. The relatively high *T*_c_ value indicates that the crystal was in the optimally-doped regime, consistent with the cobalt content measured.

The electro-transport measurements were performed on a Quantum Design Physical Property Measurement System (PPMS-9). We adopted a van der Pauw four-terminal configuration [31] for all the measurements, including longitudinal *I* − *V* curves and transverse voltages. The crystal was carefully cleaved and cut into a squared specimen with a side length of *L* = 1.14 mm and thickness of *t* = 0.024 mm. Gold wires were attached with silver paste onto the four corners (A, B, C, and D), as shown in the left inset of Figure 1. The longitudinal resistance was obtained by *R*_xx_ = (*R*_DC/AB_ + *R*_BC/AD_)/2, where *R*_DC/AB_ (*R*_BC/AD_) equals to the potential *V*_DC_ (*V*_BC_) divided by the current *I*_AB_ (*I*_AD_) using ac transport option with a frequency of 13 Hz. The normal-state resistivity above *T*_c_ was *ρ*_n_ = π*tR*_xx_/ln2 ~ 0.09 mΩ cm, consistent with the previous report [28]. The data of *I* − *V*_xx_ characteristic were collected at a fixed temperature whose fluctuation was less than 1 mK, without any detectable heating effect during the measurement.

For the measurement of transverse voltage *V*_xy_, *V*_BD_/_AC_ and *V*_AC/BD_ were measured respectively by permutating the voltage and current electrodes [32], so that the longitudinal component due to misalignment of the diagonal electrodes can be canceled out. At zero field, one obtains $V_{\mathrm{xy}}^{0}$ by Ref. [32], $V_{\mathrm{xy}}^{0}$ = (*V*_BD/AC_ − *V*_AC_/_BD_)/2. Obviously, $V_{\mathrm{xy}}^{0}$ has nothing to do with Hall effect because no magnetic field is applied. It is a non-Hall-type transverse voltage. Under external magnetic fields, similarly, we have two “branches” of the transverse voltage with the field up (*H*+) and down (*H*−), respectively, $V_{\mathrm{xy}}^{H+}$= ($V_{\mathrm{BD};\mathrm{AC}}^{H+}$−$V_{\mathrm{AC};\mathrm{BD}}^{H+}$)/2;$V_{\mathrm{xy}}^{H-}$= ($V_{\mathrm{BD};\mathrm{AC}}^{H-}$−$V_{\mathrm{AC};\mathrm{BD}}^{H-}$)/2. In most cases, $V_{\mathrm{xy}}^{H+}$ and $V_{\mathrm{xy}}^{H-}$ are mutually antisymmetric, and the conventional Hall voltage can be obtained by $V_{\mathrm{xy}}^{\mathrm{Hall}}$ = ($V_{\mathrm{xy}}^{H+}$−$V_{\mathrm{xy}}^{H-}$)/2. When the antisymmetry is broken for some reason, a non-Hall transverse signal $V_{\mathrm{xy}}^{nH}$ can be extracted by canceling out the external field effect, $V_{\mathrm{xy}}^{nH}$ = ($V_{\mathrm{xy}}^{H+}$+$V_{\mathrm{xy}}^{H-}$)/2.

## 3. Data Analysis

To study BKT dynamics in superconductors, there are two main approaches, the “conventional” approach and the dynamic scaling analysis [33]. In the conventional approach, the following signatures in transport properties in the *I* → 0 A limit are often used to recognize a BKT transition. (1) Within a temperature region slightly above *T*_BKT_, the Ohmic longitudinal resistance has a unique temperature dependence [34],

(1) $$R_{\mathrm{xx}}(T)/R_{n}\propto \exp\{-2{\left[b(T_{c}^{MF}-T_{BKT})/(T-T_{BKT})\right]}^{1/2}\}$$

where *R*_*n*_ is the normal-state resistance and *b* is a dimensionless parameter. The mean-field superconducting transition temperature $T_{c}^{\mathrm{MF}}$ is generally set to the midpoint temperature $T_{c}^{\mathrm{mid}}$ (close to the inflection temperature) [9,12,14], which is about 24.70 K. The above exponential behavior is in contrast to that of the paraconductivity effect (due to amplitude fluctuations), which shows a power-law divergence [35]. (2) The isothermal current-voltage relation around *T*_BKT_ obeys a power law $V\propto I^{\alpha }$ at low currents, which differs from the exponential dependence for vortex motion, owing to flux depinning [36]. (3) The exponent *α*(*T*) has a “universal jump” from 1 to 3 upon approaching *T*_BKT_ from above. Such a universal jump is regarded as the hallmark of BKT transition. In the BKT theory, *α*(*T*) is proportional to the superfluid density, therefore, the jump in *α*(*T*) means discontinuity in superfluid density [37], which is called the Nelson–Kosterlitz jump in literature.

According to Equation (1), *R*/*R*_n_ in logarithmic scale is plotted as a function of $(T-T_{BKT}{)}^{-1/2}$ in Figure 1. A linear dependence is shown in between $T_{c}^{\mathrm{zero}}$ and 24.81 K with the fitted parameters *T*_BKT_ = 24.42 ± 0.01K and *b* = 2.10 ± 0.01. Note that the *T*_BKT_ is very close to the zero-resistance temperature $T_{c}^{\mathrm{zero}}$, similar to previous reports in cuprate systems [9,10,11,12]. For $T_{c}^{\mathrm{mid}}$<T<$T_{c}^{\mathrm{onset}}$, however, paraconductivity effect usually becomes dominant. However, we were not able to fit the *R*(*T*) data in this region to either 2D or 3D forms of Aslamazov–Larkin theory [35]. This suggests a dimensional crossover and/or robustness of phase fluctuations in the range of $T_{c}^{\mathrm{mid}}$<T< $T_{c}^{\mathrm{onset}}$.

**Figure 2:**
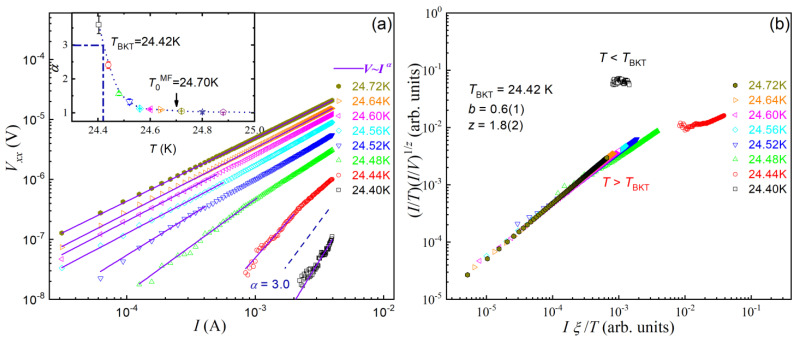
(**a**) A log–log plot of voltage-current characteristics of Ba(Fe_0.914_Co_0.086_)_2_As_2_ crystal at temperatures spanning the critical region from 24.40 K to 24.72 K. The inset shows temperature dependence of fitted by *V* ∝ *I*^α^ with the low-current data. (**b**) Dynamic scaling of all the *I* − *V* data in (**a**) according to Equation (2) in the text.

The isothermal *I* − *V* characteristics at *T* = 24.40–24.72 K with *I* = 0.03–4 mA are displayed in a log–log plot shown in Figure 2a. The linearity confirms the expected power–law relation. The exponent *α*, represented by the slope, changes with temperature. For *T* > 24.56 K, the *α* value is close to 1.0, namely, the *I* − *V* curves are basically Ohmic. When approaching *T*_BKT_, *α* increases abruptly, and it goes to 3.6 ± 0.3 at 24.40 K. The inset of Figure 2a clearly shows a jump with *α* = 3.0 ± 0.2 at *T*_BKT_, which suggests the characteristic Nelson–Kosterlitz jump anticipated for a BKT transition.

Strictly speaking, the above approach is valid only in the limit *I* → 0 A. Thus it is necessary to perform a dynamic scaling analysis, which also holds for finite currents [33]. According to the FFH theory [29], the scaling form for a 2D superconductor can be written as [33,38],

(2) $$\left(I/T){\left(I/V\right)}^{1/z}=P_{\pm }(I\xi_{\pm }/T\right)$$

where *z* is the dynamic exponent, *P*_+(−)_ is the scaling function for temperature above (below) *T*_BKT_, and $\xi_{+}\sim \exp[b(T_{c}^{MF}-T)/(T_{}^{}-T_{BKT}{)]}^{1/2}$($\xi_{-}\sim \exp[b(T_{c}^{MF}-T_{BKT})/2\pi (T_{BKT}{}_{}^{}-T_{}{)]}^{1/2}$) is the correlation length above (below) *T*_BKT_.

Figure 2b plots *Iξ*/*T* vs. (*I*/*T*)(*I*/*V*)^1/*z*^, according to Equation (2). By setting the afore-determined *T*_BKT_ = 24.42 K, and with the fitted parameters *b* = 2.1 ± 0.1 and *z* = 1.8 ± 0.2, all the *I* − *V* data points in Figure 2a basically fall onto two branches of the scaling curves (although the branch for *T* < *T*_BKT_ are limited to one set of *I* − *V* data with *T* = 24.40 K). Since the critical *I* − *V* curve follows *V* ∝*I^z^*^+1^ at *T*_BKT_, the exponent is 2.8 ± 0.2 at the BKT transition, consistent with the result of above conventional approach. Besides, the value of parameter *b* is the same with that extracted by fitting *R*/*R*_n_ with Equation (1). Therefore, the FFH dynamic scaling analysis also suggests a BKT phase transition in Ba(Fe_0.914_Co_0.086_)_2_As_2_ crystals.

As we know, BKT transition is driven by the unbinding of vortex–antivortex pairs. Below *T*_BKT_, the thermally exited vortices are in pairs because of the attractive interaction. At *T*_BKT_, the vortex pairs start to unbind, and free vortices are generated due to the contribution of entropy to the free energy. It is the unbound free vortices that contribute to the nonzero longitudinal resistance expressed by Equation (1). Interestingly, such free vortices are able to induce an abnormal nonzero $V_{\mathrm{xy}}^{0}$, like the case in CuSCs [18,19]. So probing $V_{\mathrm{xy}}^{0}$ may supply further evidence for the BKT transition.

**Figure 3:**
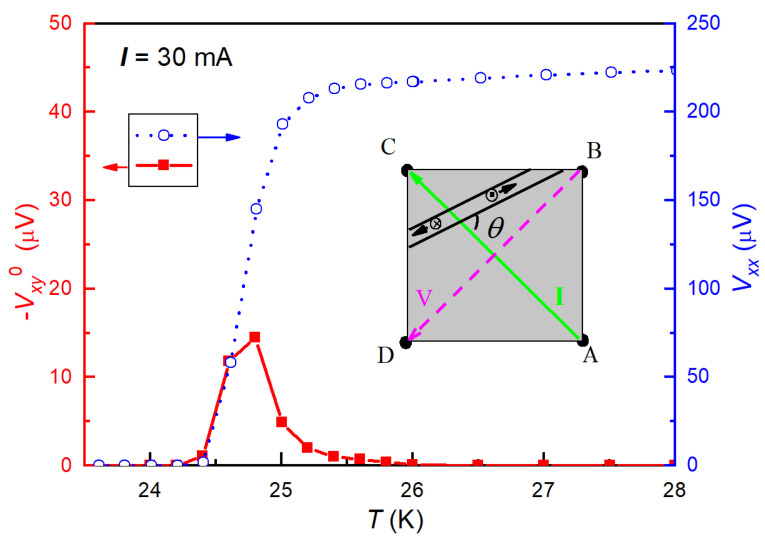
Temperature dependence of the zero-field transverse (left axis) and longitudinal (right axis) voltages for Ba(Fe_0.914_Co_0.086_)_2_As_2_ crystals. The transverse voltage was obtained by $V_{\mathrm{xy}}^{0}$ = (*V*_BD/AC_ − *V*_AC/BD_)/2, and the inset depicts the guided motion of an unbound vortex pair as well as the configuration for the measurement of *V*_BD/AC_.

Figure 3 shows $V_{\mathrm{xy}}^{0}$ as a function of temperature in Ba(Fe_0.914_Co_0.086_)_2_As_2_ crystals. The $V_{\mathrm{xy}}^{0}$ value is virtually zero in the normal state ($T>T_{c}^{\mathrm{onset}}$) and the superconducting state ($T<T_{c}^{\mathrm{zero}}$). A nonzero peak-like $V_{\mathrm{xy}}^{0}$ appears exactly within the region of superconducting transition. The maximum of $V_{\mathrm{xy}}^{0}$ is located around the midpoint of the resistive transition. It is noted that the sign and the value of the maximal $V_{\mathrm{xy}}^{0}$ depends on the electrode configuration with respect to the sample orientations. For instance, when the sample is turned over, $V_{\mathrm{xy}}^{0}$ just changes the sign. Another feature of the nonzero $V_{\mathrm{xy}}^{0}$ is that the left side of the $V_{\mathrm{xy}}^{0}$ peak coincides well with the longitudinal signal, i.e., $V_{\mathrm{xy}}^{0}$ is basically proportional to *V*_xx_ for $T_{c}^{\mathrm{zero}}$ < *T* < $T_{c}^{\mathrm{mid}}$.

The non-Hall-type transverse voltage in the absence of external magnetic field at superconducting transition was explained by the guided motion of thermally excited vortices [18,19,39]. In Ba(Fe_1−*x*_Co*_x_*)_2_As_2_, the guided motion of vortices (or in other words, with anisotropic flux pinning) are supported by the in-plane anisotropy [40] and stripe-like STM image [41]. Assuming a simple situation that fluxons can only move along the guiding direction in an angle *θ* with respect to the current A → C as shown in the inset of Figure 3, according to Reference [42], the fully guided motion of vortices generates not only longitudinal electric field,

(3) $$E_{//}^{AC}=n_{f}\Phi_{0}(F_{L}\sin^{2}\theta -F_{p}\sin\theta )/\eta c$$

but transverse electric field also,

(4) $$E_{⊥}^{BD}=n_{f}\Phi_{0}(F_{L}\sin\theta \cos\theta -F_{p}\cos\theta )/\eta c$$

where *n_f_* refers to sheet density of free vortices, Φ_0_ is flux quantum, *F*_p_ is the weak pinning force along the guiding direction, *η* is the damping coefficient of vortex motion, and *c* is the speed of light. When permutating the voltage and current, the angle between the guided motion and current turns out to be (π/2 + *θ*), and the transverse field due to current B → D becomes,

(5) $$E_{⊥}^{AC}=n_{f}\Phi_{0}(F_{L}\sin\theta \cos\theta -F_{p}\sin\theta )/\eta c$$

Since *V* = *Ed* (*d* is the length of the diagonal of the sample), and $V_{\mathrm{xy}}^{0}$ = (*V*_BD/AC_ − *V*_AC/BD_)/2, $V_{\mathrm{xy}}^{0}$ measured should be,

(6) $$V_{\mathrm{xy}}^{0}=\sqrt{2}dn_{f}\Phi_{0}F_{p}\sin(\theta -\pi /4)/\eta c$$

Obviously, $V_{\mathrm{xy}}^{0}$ is nonzero as long as *θ* ≠ (*k* +1/4)π (*k* is an integer) in the presence of free vortices. The sinusoidal variation on *θ* qualitatively agrees with our experimental observation that $V_{\mathrm{xy}}^{0}$ depends on the electrode configuration with respect to the sample orientations. When the sample is turned over, and the same electrode configuration is kept, *θ* changes into (π/2 − *θ*). Equation (6) gives $V_{\mathrm{xy}}^{0}|{}_{\theta \to (\pi /2-\theta )}=-V_{\mathrm{xy}}^{0}$, which exactly meets the experimental observation. In addition, Equations (3) and (6) explain the coincidence of longitudinal and transverse signals in Figure 3, because both are proportional to *n_f_*. At $T\geq T_{c}^{\mathrm{mid}}$, *n_f_* decreases rapidly since the superconducting Cooper pairs dissociate. This explains the drop in $V_{\mathrm{xy}}^{0}$ above $T_{c}^{\mathrm{mid}}$. The nonzero $V_{\mathrm{xy}}^{0}$ is extended to 26.0 K, suggesting superconducting phase fluctuations above *T*_c_, such as the case in cuprate superconductors [20].

**Figure 4:**
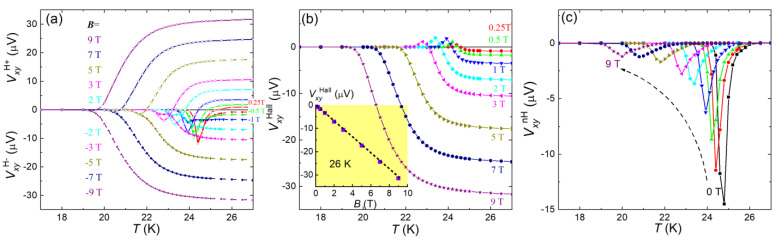
(**a**) Transverse voltages $V_{\mathrm{xy}}^{H+}$ and $V_{\mathrm{xy}}^{H-}$ at different directions of external magnetic fields as functions of temperature for the Ba(Fe_0.914_Co_0.086_)_2_As_2_ crystals. (**b**) and (**c**) plot the temperature dependence of Hall and non-Hall transverse voltages, respectively. See the experimental method in the text for details.

We also measured the transverse voltage under external magnetic fields. Figure 4a shows temperature dependence of $V_{\mathrm{xy}}^{H+}$ and $V_{\mathrm{xy}}^{H-}$ defined in the experimental paragraph. In the normal state above $T_{c}^{\mathrm{onset}}$,$V_{\mathrm{xy}}^{H+}$ and $V_{\mathrm{xy}}^{H-}$ are mutually antisymmetric with respect to the applied field, consistent with usual Hall effect. The Hall voltage $V_{\mathrm{xy}}^{\mathrm{Hall}}$, obtained by $V_{\mathrm{xy}}^{\mathrm{Hall}}$= ($V_{\mathrm{xy}}^{H+}$−$V_{\mathrm{xy}}^{H-}$)/2, is shown in Figure 4b. Indeed, $V_{\mathrm{xy}}^{\mathrm{Hall}}$ increases linearly with increasing field in the normal state (shown in the inset). The value and sign of $V_{\mathrm{xy}}^{\mathrm{Hall}}$ is consistent with previous reports [28]. At the superconducting transition, an anomalous sign reversal was observed, similar to previous study on Ba(Fe_0.9_Co_0.1_)_2_As_2_ crystal [43]. Here we emphasize that this anomalous sign reversal is related to applied fields, as it changes sign upon field reversal. Further discussion on its origin is beyond the scope of this paper.

At the superconducting transition, however, the transverse voltage does not change sign upon reversal of magnetic field, especially under low magnetic fields. So, there exists a non-Hall-type signal $V_{\mathrm{xy}}^{\mathrm{nH}}$, which is shown in Figure 4c. Similar to the $V_{\mathrm{xy}}^{0}$ signal shown in Figure 3, $V_{\mathrm{xy}}^{\mathrm{nH}}$ also exhibits a peak at the superconducting transition, and the peak height decreases systematically with increasing magnetic field, which implies that $V_{\mathrm{xy}}^{\mathrm{nH}}$, together with $V_{\mathrm{xy}}^{0}$, comes from the same origin. If $V_{\mathrm{xy}}^{\mathrm{nH}}$ is due to the guided motion of vortices, as was earlier discussed in Na-Ta foils [42], the decrease in $V_{\mathrm{xy}}^{\mathrm{nH}}$ can be qualitatively explained by the decrease of anisotropic pinning under magnetic fields (the pinning force perpendicular to the vortex-guided direction is reduced by the increasing number of vortices (due to the increasing magnetic field), and the vortices may easily slip along this direction, which smears out the anisotropic pinning effect). Here we should mention an alternative explanation for the non-Hall voltages in terms of asymmetric inhomogeneity [44]. However, even if some unavoidable minor asymmetric inhomogeneity plays a role for the non-Hall signal, it cannot bring about the BKT dynamics with *α*(*T*) = 3 at *T*_BKT_ by itself. Besides, numerical simulations [45] indicate that inhomogeneity in *T*_c_ merely broadens the BKT transition without changing the universality class (*z* = 2), which agrees with our experimental observations.

## 4. Summary and Discussion

The possible appearance of BKT-type phase transition in Ba(Fe_1−*x*_Co*_x_*)_2_As_2_ bulk crystals suggests a quasi-2D nature for iron-based superconductivity. Indeed, 2D antiferromagnetic spin fluctuations, which are mostly believed to be the glue of Cooper pairing, were revealed in BaFe_1.84_Co_0.16_As_2_ by neutron scattering experiment [46]. The 2D nature of superfluid density was found in Li(C_5_H_5_N)_0.2_Fe_2_Se_2_ superconductor [47]. Recently, a 2D-like (or BKT-like) nature in organic ion intercalated FeSe superconductors (TBA)_x_FeSe is also supported by both anisotropic transport and *I* − *V* curves [48]. Furthermore, the observation of high-temperature superconductivity in FeSe monolayer grown on SrTiO_3_ substrate [49] directly suggests 2D superconductivity in FeSCs. The thickness of the FeSe monolayer is only about 2.8 Å, which means that the coherence length perpendicular to the layers, *ξ*_c_, is shorter than 2.8 Å. Therefore, it is not so surprising that 2D superconducting behavior was manifested in Ba(Fe_1−*x*_Co*_x_*)_2_As_2_, because the FeAs interlayer spacing is about 6.5 Å [28]. Here we point out that the conventional estimation of *ξ_c_* from the anisotropy ratio in *H*_c2_ [50] using $\xi_{\mathrm{ab}}/\xi_{c}=H_{c2}^{//}/H_{c2}^{⊥}$, which gives *ξ_c_* ~ 25 Å, may be misleading. This is because the measured *H*_c2_ values are basically Pauli-limited (rather than orbital-limited) and FeSC are multi-band superconductors. For a specific superconducting pairing channel, *ξ_c_* could be significantly smaller than the simple estimated from *H*_c2_.

To summarize, we have presented the possible evidence for BKT phase transition in a typical FeSC Ba(Fe_0.914_Co_0.086_)_2_As_2_. The observation of non-Hall transverse voltage, probably caused by the guided motion of thermally activated vortices, in turn, further indicates the BKT scenario with vortex–antivortex unbinding. Our results suggest that, similarly to CuSCs, two-dimensionality also plays an important role for high-temperature superconductivity in iron pnictides.

## Data Availability

The datasets used and/or analyzed during the current study are available from the corresponding author on reasonable request.
